# Reconstruction of an Open Achilles Tendon Rupture With a Large Soft Tissue Defect Using an Achilles Tendon Allograft and Distally Based Sural Artery Neurocutaneous Flap: A Case Report

**DOI:** 10.7759/cureus.78360

**Published:** 2025-02-01

**Authors:** Cheng-Hao Tai, Yi-Chen Li, Tsung-Chiao Wu, Kai-Chiang Yang, Chen-Chie Wang

**Affiliations:** 1 School of Medicine, Tzu Chi University, Hualien, TWN; 2 Department of Orthopedic Surgery, Taipei Tzu Chi Hospital, Buddhist Tzu Chi Medical Foundation, New Taipei City, TWN; 3 Graduate Institute of Biomedical Materials and Tissue Engineering, Taipei Medical University, New Taipei City, TWN; 4 Precision Medicine Ph.D. Program, National Tsing Hua University, Hsinchu, TWN; 5 School of Dental Technology, College of Oral Medicine, Taipei Medical University, Taipei City, TWN; 6 Department of Orthopedics, School of Medicine, Tzu Chi University, Hualien, TWN

**Keywords:** graft integration, open achilles tendon rupture, soft tissue coverage, sural artery neurocutaneous flap, tendon allograft

## Abstract

Achilles tendon ruptures are categorized as acute or chronic based on the timing of diagnosis, with chronic ruptures presenting considerable surgical challenges. This case report details the management of a 58-year-old man who presented with a chronic open Achilles tendon rupture accompanied by a large soft tissue defect. The injury, caused by a sheet metal cutting accident, was further complicated by wound necrosis and infection. Surgical management involved reconstruction of the Achilles tendon using an allograft, along with defect coverage using a distally based sural artery neurocutaneous flap. Postoperative outcomes were favorable, with marked improvements in both pain and functional capacity. At six months postoperatively, the patient exhibited complete graft integration, satisfactory functional recovery, and a return to daily activities without signs of ankle instability. This case demonstrates the efficacy of tendon allograft reconstruction combined with vascularized flap coverage in managing complex Achilles tendon injuries with extensive tissue loss. Further research is warranted to evaluate the long-term success of this approach.

## Introduction

Achilles tendon ruptures are categorized as acute or chronic on the basis of the time of diagnosis. Chronic Achilles tendon ruptures refer to injuries diagnosed more than four to six weeks after the initial insult, whereas acute ruptures refer to injuries diagnosed within the first four weeks [1].

Acute Achilles tendon ruptures are typically managed through open repair or minimally invasive surgery [2-4]. By contrast, chronic Achilles tendon ruptures present considerable surgical challenges, particularly in selecting reconstruction strategies that account for defect size and tissue viability. For defects smaller than 2 cm, primary end-to-end repair is usually sufficient [5]. For defects measuring between 2 and 5 cm, methods such as V-Y lengthening and flexor hallucis longus tendon transfer are commonly employed [5]. Larger defects exceeding 5 cm necessitate more complex approaches, including fascia turndown advancements and tendon transfers, often incorporating autografts or allografts to optimize functional outcomes [5].

In cases involving open Achilles tendon ruptures with large soft tissue defects, commonly employed coverage strategies include the use of the free anterolateral thigh (ALT) flap and sural flap [6-8]. Although these strategies effectively address soft tissue coverage, they fail to resolve tendon reconstruction; consequently, patients remain at risk of impaired or complete loss of tendon function. Additionally, the use of the ALT flap requires a large donor tissue and often necessitates secondary debulking procedures, further increasing the risk of postoperative complications [7].

## Case presentation

A 58-year-old man with a history of type 2 diabetes mellitus sustained a right Achilles tendon rupture with an open wound caused by a cutting injury from sheet metal while tidying his garden. Initially, he was treated at another hospital, where he underwent Achilles tendon repair following wound irrigation and local debridement. However, poor wound healing and skin necrosis were noted, necessitating two additional debridement procedures. Despite these efforts, the wound exhibited persistent purulent discharge, prompting the patient to seek a second opinion at our hospital. Upon thorough examination, the patient underwent further debridement at our institution. Wound care included normal saline wet dressings every eight hours and intravenous oxacillin administration. Subsequent wound cultures revealed an Enterobacter infection, necessitating a switch to intravenous moxifloxacin.

**Figure 1:**
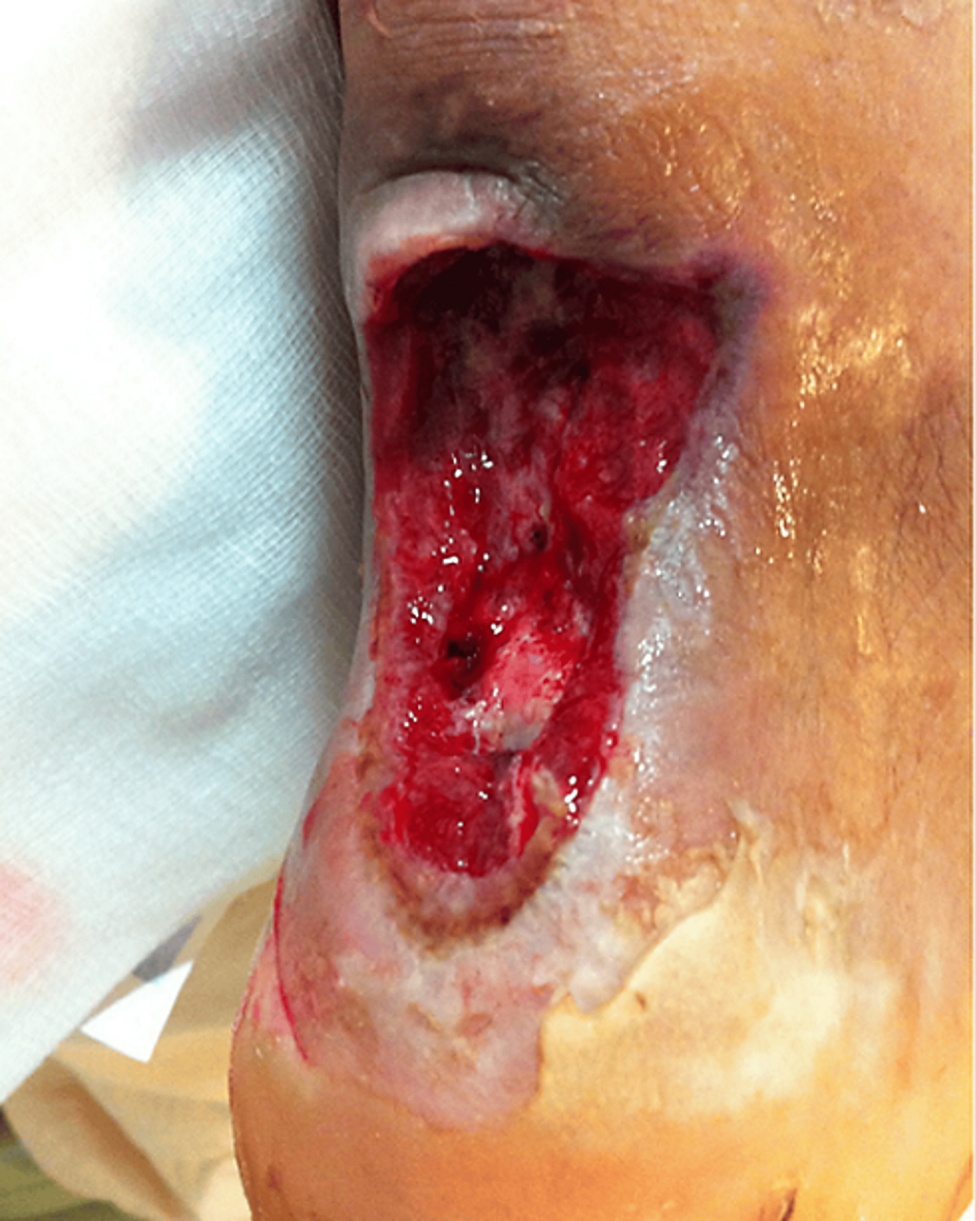
Post-debridement wound Post-debridement wound, measuring 6 × 7 cm^2^, after two surgical procedures at our hospital.

After two debridement procedures, the wound, measuring 6 × 7 cm², appeared clean without purulent discharge (Figure 1).

**Figure 2:**
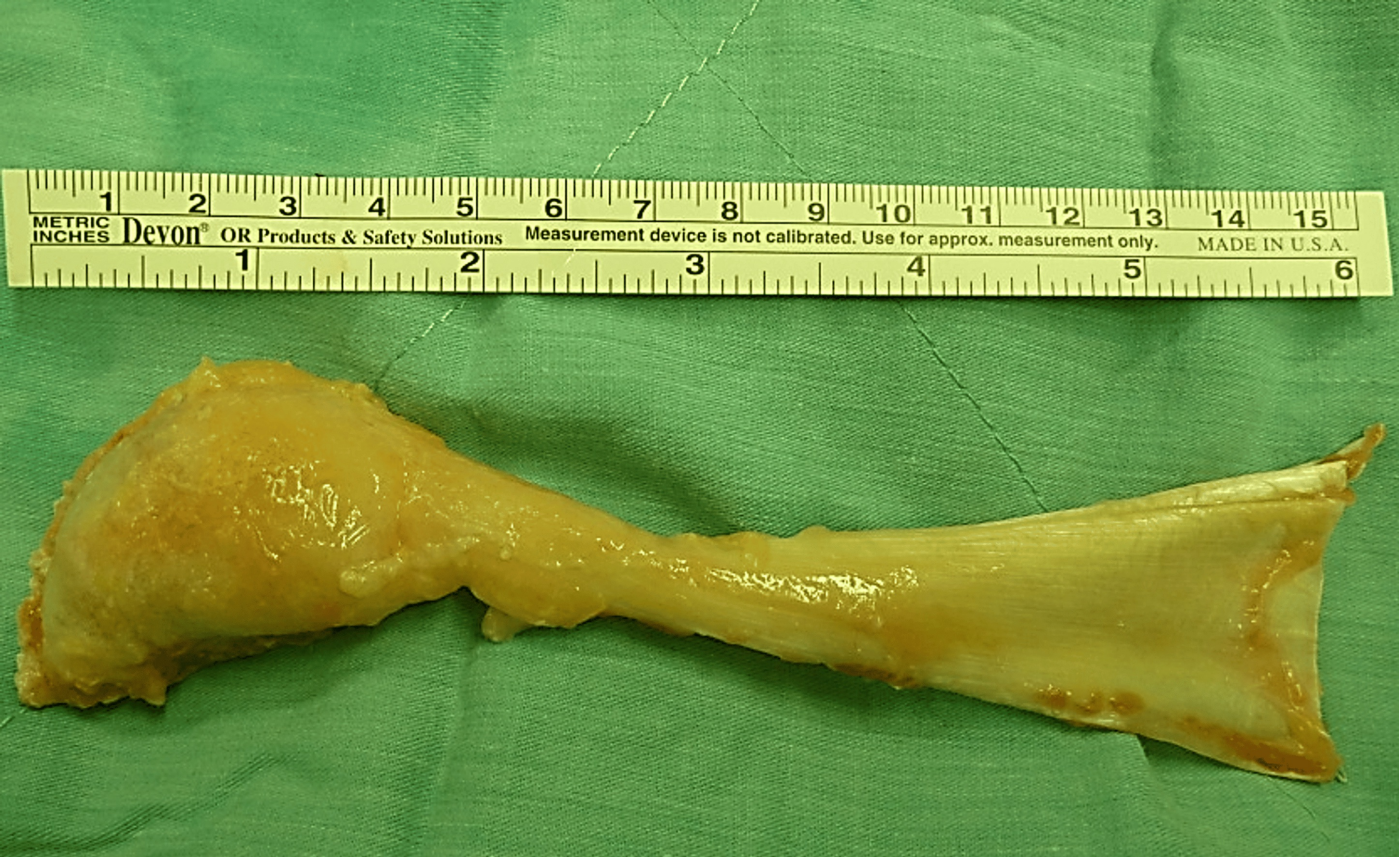
Achilles tendon allograft Achilles tendon allograft, including its calcaneal bony portion, prepared for reconstruction.

Five weeks after the initial injury, the patient underwent Achilles tendon allograft reconstruction combined with coverage using a distally based sural artery neurocutaneous flap and split-thickness skin grafting for the flap donor site. The Achilles tendon allograft was obtained from our tissue bank and preserved in a fresh-frozen state (Figure 2).

**Figure 3:**
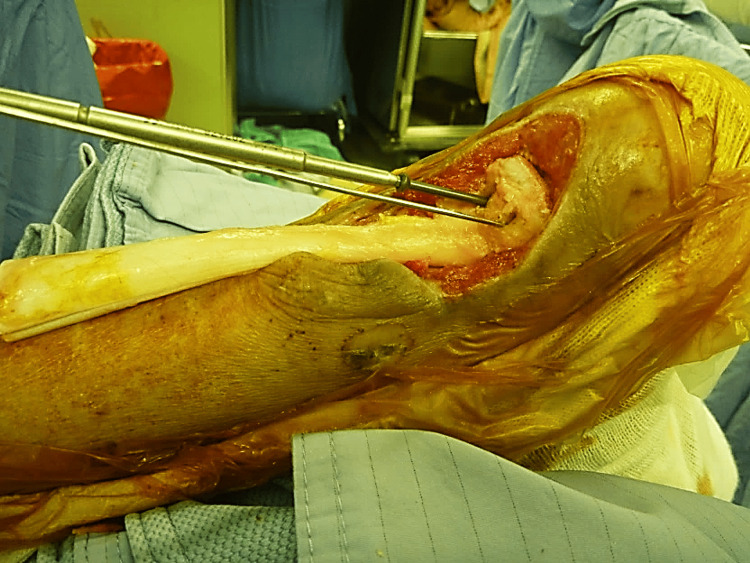
Placement of the bony portion of allograft Placement of the bony portion of allograft into the calcaneal bone trough, which was secured with two screws.

During the surgery, a calcaneal bone trough was created at the calcaneal tuberosity. The allograft’s calcaneal portion was shaped to match the trough and inserted using a press-fit technique (Figure 3).

**Figure 4:**
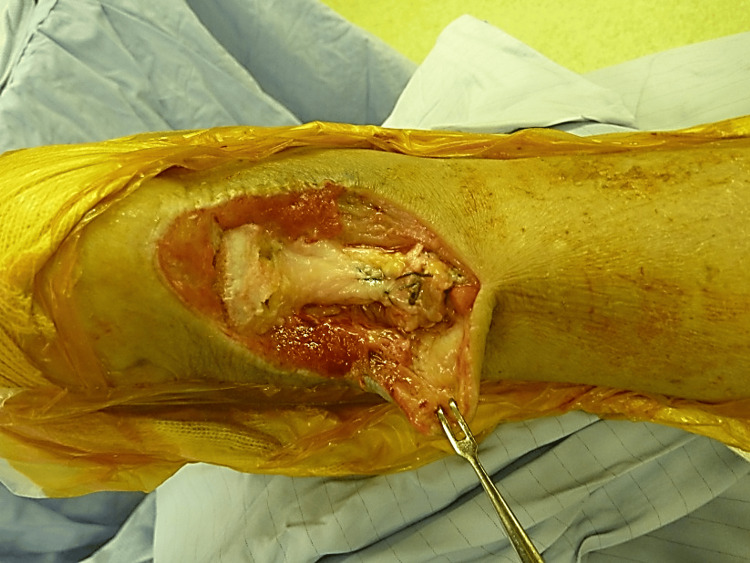
Ruptured Achilles tendon sutured to the allograft Ruptured Achilles tendon sutured to the allograft following tension adjustment at the repaired site.

Fixation was achieved using two screws, and the retracted proximal Achilles tendon stump was sutured to the allograft by using two sets of Bunnell-type suture configurations. Tension at the repair site was adjusted appropriately, and the ankle was maintained in a plantar-flexed position (Figure 4).

**Figure 5:**
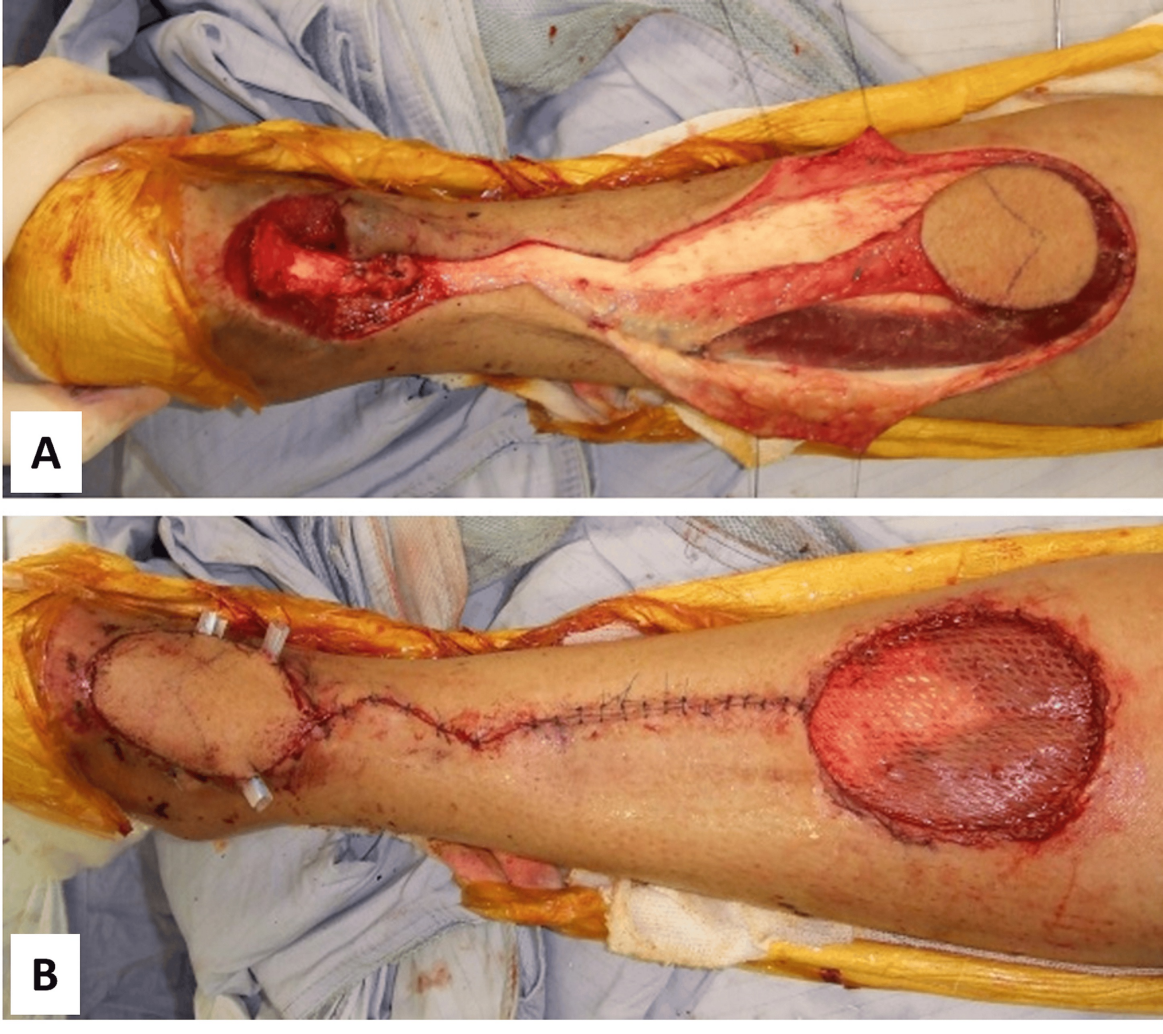
Sural artery neurocutaneous flap (A) Harvesting of the distally based sural artery neurocutaneous flap. (B) Flap rotated 180° and sutured to cover the wound.

To address the soft tissue defect, a distally based sural artery neurocutaneous flap was harvested, rotated 180°, and used for coverage (Figure 5). The flap donor site was then sealed with a split-thickness skin graft harvested from the ipsilateral anterior thigh. During the postoperative follow-up, the wound exhibited 100% graft take and satisfactory flap circulation, with only minor ecchymosis observed.

**Figure 6:**
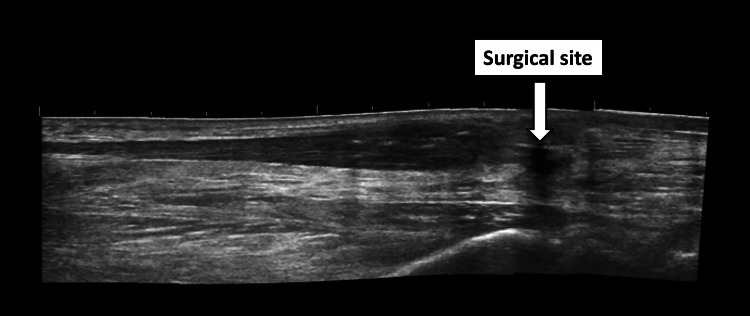
Ultrasound examination Ultrasound examination at five months postoperatively, displaying satisfactory healing of the surgical site.

At four months postoperatively, the patient began gradual weight-bearing with the assistance of a cane. By five months, an ultrasound examination confirmed satisfactory healing of the surgical site (Figure 6).

**Figure 7:**
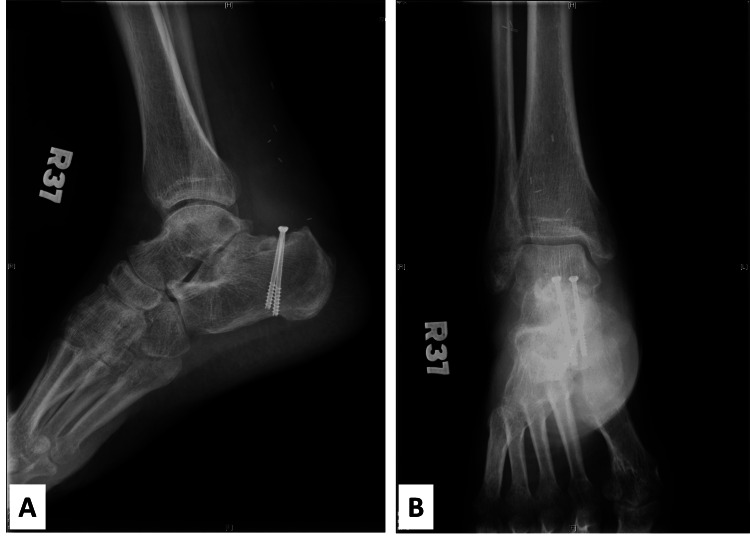
X-ray images to the recipient site X-ray images depicting adequate bone union between the recipient site and a bony portion of the allograft. (A) Lateral view. (B) Anteroposterior view.

At the six-month follow-up, X-ray imaging demonstrated complete healing at the junction between the allograft and the patient’s own bone (Figure 7).

**Figure 8:**
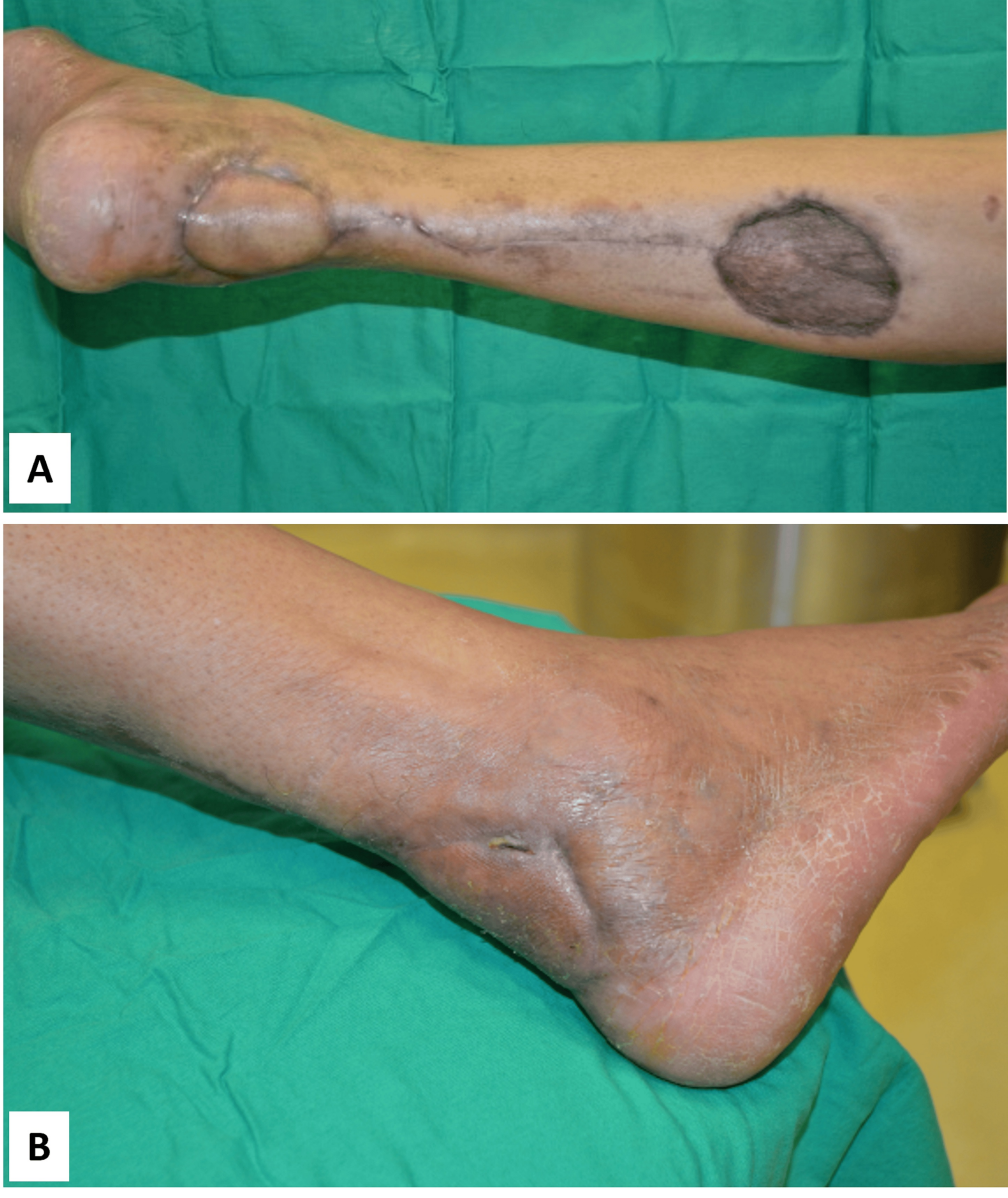
Postoperative photographs Postoperative photographs at six months, demonstrating well wound healing. (A) Posterior view. (B) Lateral view.

The wound healed well, and a negative Thompson test indicated restored Achilles tendon function (Figure 8). 

Clinical outcomes indicated successful tendon healing, with follow-up extending over several years. Pain assessment scores on the visual analog scale improved from 5 preoperatively to 1 postoperatively. The American Orthopaedic Foot and Ankle Society score increased from 43 preoperatively to 92 postoperatively, reflecting a substantial improvement in ankle function. The patient reported a full return to daily activities without ankle instability.

Mental and physical health assessments conducted using the 12-item Short Form Survey revealed significant improvements. Preoperatively, the mental component score (MCS-12) and physical component score (PCS-12) were 19.1 and 24.0, respectively. At the 10-year follow-up, the MCS-12 and PCS-12 scores increased to 60.7 and 55.3, respectively, indicating a substantial recovery in mental and physical health.

## Discussion

The Achilles tendon is essential for locomotion because it enables activities such as walking and running. Repairing Achilles tendon ruptures is crucial for restoring function and improving quality of life, allowing individuals to return to physical activities. However, open Achilles tendon ruptures accompanied by large tissue defects present substantial challenges for orthopedic surgeons [7]. Open Achilles tendon ruptures should be treated cautiously, with measures such as irrigation with massive normal saline, delicate tendon stump repair, and prolonged preventive antibiotic therapy. Nonetheless, skin necrosis with wound infection is frequently reported and often requires prolonged treatment [7]. Despite repeated wound debridement being an essential management step in such complex cases, this procedure may result in a large tendon defect without adequate soft tissue coverage, posing a serious concern for subsequent reconstruction. The challenge is compounded by an irreparable tendon gap resembling a chronic Achilles tendon rupture, further complicated by a large soft tissue and skin defect.

Chronic Achilles tendon ruptures remain a significant clinical challenge [9, 10]. Numerous reconstruction methods have been proposed based on the rupture location, gap size, and surgeon preference. For large tendon defects, tendon grafting is often necessary. Autografts are widely used due to their favorable biocompatibility and mechanical properties. However, they are associated with risks such as donor site complications, increased surgical complexity, and limited graft availability [11, 12]. Allografts offer an attractive alternative, providing several advantages, including reduced surgical time, adequate tissue quality, minimized donor site morbidity, and robust mechanical properties [13]. These advantages make allografts particularly suitable for restoring tendon function after rupture. However, challenges such as the risk of disease transmission, high costs, potential immune responses or rejection, and the need for successful integration into host tissue persist [13].

In recent decades, various synthetic materials have been investigated for chronic Achilles tendon rupture repair. These materials can preserve active tendon structures and reduce surgical morbidity. However, concerns such as infection risk, foreign body reactions, and limited biological integration with native tendon tissue restrict their application [14, 15]. These complications, including postoperative stiffness, pain, or graft failure, pose challenges to their long-term success [11, 16, 17].

To ensure sufficient blood flow to the allograft and achieve adequate soft tissue coverage, a distally based sural artery neurocutaneous flap can be combined with allograft reconstruction [18, 19]. This approach not only ensures vascular supply to the graft but also addresses potential sensory deficits in the reconstructed area [18]. Alternative soft tissue coverage methods, such as free flaps or posterior tibial artery perforator flaps, have also been proposed. However, these methods often involve longer surgical times due to the need for vascular anastomosis, donor site morbidity, and the potential requirement for secondary debulking procedures, which limit their widespread adoption.

Our study highlights the successful management of an open Achilles tendon rupture with a large tissue defect using Achilles tendon allograft reconstruction combined with a distally based sural artery neurocutaneous flap. This approach enabled successful healing and functional restoration. At six months postoperatively, the patient demonstrated substantial improvements in pain and functional capacity, allowing a return to daily activities without ankle instability.

## Conclusions

Herein, we presented a case of an open Achilles tendon rupture with a large tissue defect, treated using Achilles tendon allograft reconstruction combined with a distally based sural artery neurocutaneous flap. The patient was able to resume previous activities with a satisfactory functional outcome. This case underscores the critical role of timely diagnosis and appropriate intervention in managing chronic tendon injuries. Allograft reconstruction, particularly when supported by vascularized flaps, offers a viable and effective surgical option for addressing large defects. However, further research is needed to evaluate the long-term outcomes and broader applicability of this approach in similar cases.
